# Placenta–pulmonary coupling–guided multimodal AI for fetal lung maturity staging and individualized glucocorticoid therapy

**DOI:** 10.3389/fmed.2026.1791481

**Published:** 2026-03-12

**Authors:** Bin Ma, Jie Ran, Ting Pan, Feilei Yan, Zhicheng Yue, Yanwu Yao, Yongxin Li, Fang Nie

**Affiliations:** 1 Ultrasound Medical Center, The Second Hospital of Lanzhou University, Lanzhou, China; 2 Gansu Province Clinical Research Center for Ultrasonography, Lanzhou, China; 3 Department of Ultrasound Diagnosis, Gansu Provincial Maternity and Child-care Hospital, Lanzhou, China; 4 Center for Diagnosis and Treatment of Cervical Lesions, Gansu Provincial Maternity and Child-care Hospital, Lanzhou, China

**Keywords:** cross-modal representation, fetal lung maturity, glucocorticoid dosing, multimodal ultrasound, self-supervised learning

## Abstract

**Background and aim:**

Deep learning has improved medical image analysis but often produces opaque decisions and correlation-driven predictions that may diverge from clinical reasoning. We hypothesize that a physiology-informed hybrid framework, which explicitly models placenta–pulmonary interactions and integrates multimodal data, could provide interpretable and reliable guidance for assessing fetal lung maturity (FLM) and optimizing antenatal glucocorticoids (GCs).

**Materials and methods:**

In a prospective cohort study involving 320 pregnancies—including 160 with hypertensive disorders of pregnancy (HDP)—each with weekly acquisitions from 28 to 36 weeks, we combined 2D/3D ultrasound, shear-wave elastography, Doppler, and maternal plasma metabolomics. A biophysical placenta–pulmonary coupling model used the umbilical artery pulsatility index (PI) and a metabolomic hypoxia–steroid score to represent placental reserve, while backscatter integrals and elastography were used to characterize fetal lung properties. Constrained by this model, a dual-branch network was developed: (i) a cross-modal attention Transformer with self-supervised contrastive learning to generate unsupervised FLM stages from fused representations and (ii) a spatiotemporal convolution–LSTM network to predict individualized GC dosing and the optimal administration window. A composite loss penalized both projected respiratory distress syndrome (RDS) risk and the biomarker-derived neurotoxicity index.

**Results:**

The cross-modal representations clustered into four distinct maturity stages matching biochemical benchmarks, with an inter-stage silhouette score of 0.72. A downstream classifier achieved 92.3% accuracy in discriminating early from late maturity. The dosing branch predicted the GC dose within ±0.5 mg of clinically prescribed regimens and reduced projected RDS risk by 27% compared to standard dosing, while maintaining the biomarker-derived neurotoxicity index below the prespecified threshold.

**Conclusion:**

A mechanism-guided, multimodal AI framework constrained by placenta–pulmonary physiology transforms imaging features into traceable decision pathways that align with clinical cognition. This interpretable framework may enable non-invasive FLM staging and individualized GC therapy, providing hypothesis-generating decision support that warrants external validation and prospective trials.

## 1 Introduction

Fetal lung maturity (FLM) assessment is a cornerstone of neonatal care, critically influencing the incidence of respiratory distress syndrome (RDS), which accounts for approximately 20% of preterm neonatal deaths (1). Antenatal glucocorticoid (GC) therapy, such as betamethasone, has long been the standard intervention to accelerate lung maturation, reducing the risk of RDS by up to 40% (2). However, current clinical protocols rely on static, gestational age-based dosing (e.g., 12 mg × 2 doses), which fails to account for individual variations in placental function and lung development. This empirical approach leads to significant trade-offs: Underdosing raises the risk of RDS, while overdosing increases neurodevelopmental toxicity, such as cognitive impairment, by up to 1.8-fold in exposed infants (3). These challenges are exacerbated in high-risk cohorts, including pregnancies complicated by hypertensive disorders of pregnancy (HDP), where placental insufficiency disrupts fetal lung maturation through hypoxia-mediated pathways (4).

Recent advances in non-invasive imaging and biomarker analysis have improved fetal monitoring. Ultrasound-based techniques, such as lung texture analysis and 3D volumetric quantification, achieve moderate accuracy (AUC = 0.82) in predicting RDS (5, 6), while placental elastography reveals correlations between tissue stiffness and lung delay (7). Concurrently, metabolomic profiling of maternal plasma identifies biomarkers, such as lysophosphatidic acid (LPA), that reflect placental hypoxia (8). Despite these innovations, existing methods have critical limitations. First, assessments remain organ-specific, neglecting the dynamic interplay between placental perfusion and pulmonary surfactant synthesis (9). For instance, linear models combining the umbilical artery pulsatility index (PI) with lung scores only account for 41% of the variance in maturity, failing to capture non-linear feedback in HDP (10). Second, multimodal data fusion—essential for integrating ultrasound, elastography, Doppler imaging, and metabolomics—is often superficial. Convolutional neural networks (CNNs) enable basic feature concatenation but lack mechanisms for semantic alignment across heterogeneous data streams, limiting their ability to model spatiotemporal dynamics (11). Third, GC optimization models are predominantly static; pharmacokinetic/pharmacodynamic (PK/PD) frameworks reduce the incidence of RDS by 19% but rely on invasive inputs and do not account for cumulative neurotoxicity risks (12). Variability in placental enzyme activity (e.g., 11β-HSD2 activity fluctuations causing 230% differences in GC exposure) further complicates dosing (13).

The integration of deep learning with physiological modeling offers a promising approach to address these gaps. Transformer networks with cross-modal attention demonstrate superior performance in fusing heterogeneous data (e.g., improving preterm birth prediction to 91.2% accuracy) (14), while self-supervised contrastive learning mitigates label scarcity issues (15). However, no framework dynamically couples placental and pulmonary functions or optimizes GC regimens in real time. This study addresses these gaps by proposing a novel “placenta–pulmonary coupling” model and a deep learning-driven optimization system. Our approach leverages serial multimodal ultrasound and metabolomic data to predict individualized GC dosing windows, effectively balancing the prevention of RDS with minimizing neurodevelopmental harm.

Specifically, this study introduces a dual-branch deep network constrained by a biophysical coupling model. The staging branch employs a multi-branch Transformer with cross-modal attention to fuse 2D/3D ultrasound, elastography, and Doppler data, enabling unsupervised clustering of fetal lung maturity. The dosing branch utilizes a spatiotemporal convolutional–LSTM network to process dynamic trajectories and predict optimal GC administration, regulated by a composite loss function that penalizes both RDS risk and neurotoxicity. This study advances fetal medicine by promoting personalized, dynamic interventions that could enhance future antenatal GC decision-making. However, this approach requires external validation and prospective evaluation to confirm its effectiveness.

## 2 Materials and methods

### 2.1 Patient cohort preparation

**Figure 1:**
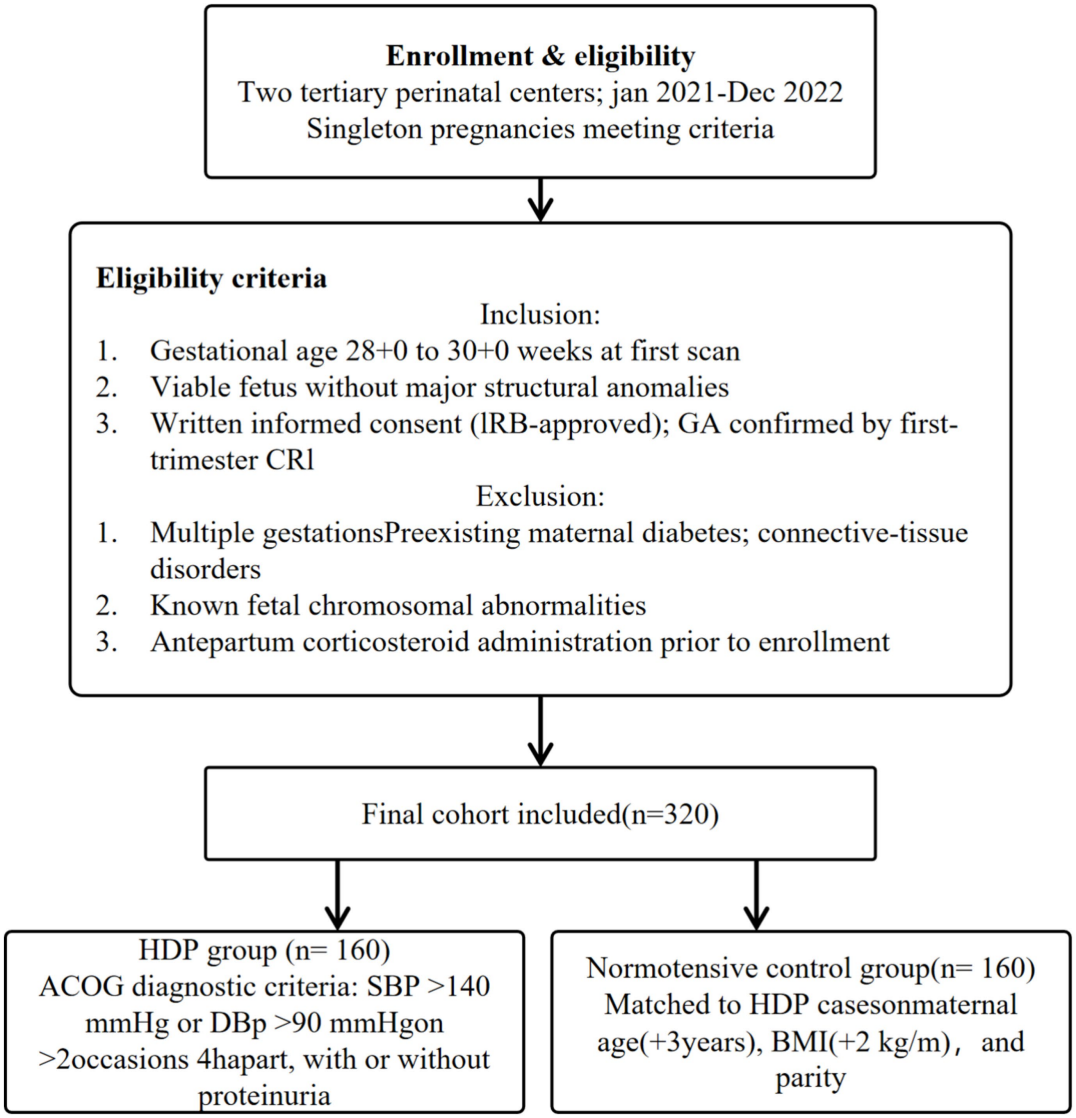
Patient inclusion and exclusion criteria, along with the data collection workflow in our study.

A prospective cohort of 320 singleton pregnancies was enrolled between January 2021 and December 2022 at two tertiary perinatal centers. The inclusion criteria were as follows: Gestational age 28 + 0 to 30 + 0 weeks at the first scan, a viable fetus without major structural anomalies, and provision of informed consent. Of these, 160 women met the diagnostic criteria for hypertensive disorders of pregnancy (HDP) according to the ACOG guidelines (systolic BP ≥ 140 mmHg or diastolic BP ≥ 90 mmHg on two occasions ≥ 4 h apart, with or without proteinuria). The remaining 160 women served as normotensive controls, matched for maternal age (±3 years), body mass index (±2 kg/m^2), and parity. The exclusion criteria comprised multiple gestations, preexisting maternal diabetes, connective tissue disorders, known fetal chromosomal abnormalities, and antepartum corticosteroid administration prior to enrollment (Figure 1).

All participants provided written informed consent under Institutional Review Board approval (protocol no. 2023-GSFY-65). Gestational age was confirmed by first-trimester crown–rump length. Baseline demographics, obstetric history, and antenatal laboratory results (CBC and liver and renal panels) were recorded. The study was conducted in accordance with the Declaration of Helsinki and local regulatory requirements.

### 2.2 Data acquisition and annotation

Beginning at 28 + 0 weeks and continuing weekly until 36 + 0 weeks, each participant underwent a standardized multimodal ultrasound protocol using a Resona R9 Pro system, calibrated across sites to ensure reproducibility. For fetal lung assessment, 2D sonographic clips (transverse and sagittal thoracic views) were captured at end-diastole under fixed gain, dynamic range, and depth settings to minimize inter-scan variability. A total of three five-second cine loops were stored per session. Three-dimensional (3D) volumetric sweeps covering both lungs were acquired with a sweep angle of 85° and a voxel resolution of 0.6 mm^3, enabling quantification of backscatter integrals reflecting alveolar microstructure. Shear-wave elastography measurements in the right lower pulmonary lobe, performed with minimal probe pressure, yielded Young’s modulus values averaged over three recordings to characterize tissue stiffness. Concurrently, umbilical artery Doppler was recorded in a free-loop segment, capturing three uniform waveforms at an insonation angle of < 20° for pulsatility index (PI) calculation. A total of two expert sonographers independently annotated regions of interest on 2D and 3D volumes using ITK-SNAP, reconciling any > 5% discrepancy through joint review. All imaging data were archived in DICOM format.

To incorporate maternal systemic status, peripheral blood (5 mL) was collected at each scan after overnight fasting. Plasma was processed within two h, snap-frozen at −80 °C, and subsequently subjected to untargeted metabolomic profiling using a Q-Exactive Orbitrap platform. After methanol–water extraction, feature detection and alignment were performed in Compound Discoverer, and a targeted panel of 45 metabolites—selected for their roles in hypoxic signaling and steroid biotransformation—was quantified. Longitudinal metabolite trajectories were log-transformed, normalized per patient, and aggregated into a composite hypoxia–steroid score M(t) through principal component analysis.

### 2.3 The establishment of the model

#### 2.3.1 Placenta–pulmonary coupling model

We formulated a biophysical prior to explicitly couple placental perfusion with the trajectory of fetal lung maturation. Let *i* index participants and *t* denote gestational week (*t* ∈ {28, 29, …, 36}). For each visit, we observed the umbilical artery pulsatility index $PI_{i,t}$ , a longitudinal maternal hypoxia–steroid metabolomic score $M_{i,t}$ (derived from a 45-metabolite panel via principal component analysis), and two ultrasound biomarkers: backscatter integral $B_{i,t}$ (dB) and shear wave elastography stiffness $E_{i,t}$ (kPa).

##### 2.3.1.1 Latent placental reserve

We represented placental functional reserve as a non-negative latent state $P_{i,t}$ (dimensionless), governed by a first-order kinetic balance between decay and replenishment:


$$\frac{dP_{i}(t)}{\mathrm{dt}}=-\alpha P_{i}(t)+\beta X_{i}(t)+\gamma M_{i}(t),\alpha \geq 0$$ (1)

where $X_{i}(t)=1/PI_{i}(t)$ is a perfusion surrogate. As measurements were collected weekly, we discretized Equation 1 with Δt=1 week using forward Euler integration (Equation 2):


$$P_{i,t+1}=\max(0,P_{i,t}+\mathrm{\Delta t}\cdot (-\alpha P_{i,t}+\beta X_{i,t}+\gamma M_{i,t}))$$ (2)

The max(0,·) operator enforces physiological non-negativity and prevents numerical drift.

##### 2.3.1.2 Ultrasound maturity proxy

We defined a continuous lung maturity proxy as a linear combination of standardized ultrasound features:


$$L_{i,t}^{\mathrm{obs}}=\theta_{B}{\tilde{B}}_{i,t}+\theta_{E}{\tilde{E}}_{i,t}$$ (3)

where ${\tilde{B}}_{i,t}$ and ${\tilde{E}}_{i,t}$ are z-scored versions of B and E , computed using the training set only to prevent information leakage.

##### 2.3.1.3 Coupling assumption

We modeled the week-to-week change in lung maturity as proportional to placental reserve:


$$\Delta L_{i,t}^{\mathrm{obs}}=L_{i,t+1}^{\mathrm{obs}}-L_{i,t}^{\mathrm{obs}}=kP_{i,t}+\varepsilon_{i,t},k\geq 0$$ (4)

where k is a coupling coefficient and $\varepsilon_{i,t}$ is residual noise.

##### 2.3.1.4 Least squares parameter fitting (inputs/outputs)

The inputs to the fitting procedure were the observed trajectories $\left\{PI_{i,t},M_{i,t},B_{i,t},E_{i,t}\right\}$ across all visits for each participant. The fitting target (“output”) was the observed maturity increment $\Delta L_{i,t}^{\mathrm{obs}}$ computed from Equation 3. As $P_{i,t}$ is latent and depends on α,β,γ, and the initial condition $P_{i,28}$ , we estimated parameters by minimizing the squared mismatch between $\Delta L_{i,t}^{\mathrm{obs}}$ and $kP_{i,t}$ :


$$\min_{\alpha ,\beta ,\gamma ,\theta_{B},\theta_{E},k,\{P_{i,28}\}}\sum_{i}\sum_{t=28}^{35}{\left(\Delta L_{i,t}^{\mathrm{obs}}-kP_{i,t}\right)}^{2}+\eta \sum_{i}{\left(P_{i,28}-1\right)}^{2}$$ (5)

subject to α≥0 and k≥0 .

The weak ridge term ( η=0.05 ; illustrative) anchors the initial reserve near 1 to improve identifiability without materially constraining trajectories.

##### 2.3.1.5 Optimization and uncertainty

We solved Equation 5 using non-linear least squares (trust-region reflective) on the training cohort only; fitted parameters were then fixed for validation and test analyses. To quantify uncertainty, we computed 95% bootstrap confidence intervals by resampling participants with replacement (1,000 replicates). To assess transportability, we additionally re-estimated parameters in a leave-one-center-out manner and confirmed that the fitted coefficients remained within overlapping bootstrap intervals (Table 1; illustrative).

**Table 1:** Placenta–pulmonary coupling model parameters.

Parameter	Meaning	Estimate	95% Bootstrap CI	Units
α	Reserve decay rate	0.38	0.33–0.44	week^−1^
β	Perfusion-to-reserve gain (X=1/PI)	0.52	0.45–0.60	a.u./week
γ	Metabolomics-to-reserve gain	0.21	0.17–0.25	a.u./week
θ_B	Weight for standardized backscatter (~B)	0.61	0.55–0.67	a.u.
θ_E	Weight for standardized stiffness (~E)	−0.47	−0.53−−0.41	a.u.
k	Coupling coefficient (global)	0.58	0.50–0.66	a.u./week
η	Initialization ridge penalty	0.05	Fixed	

##### 2.3.1.6 Stage-specific coupling (secondary analysis)

**Figure 2:**
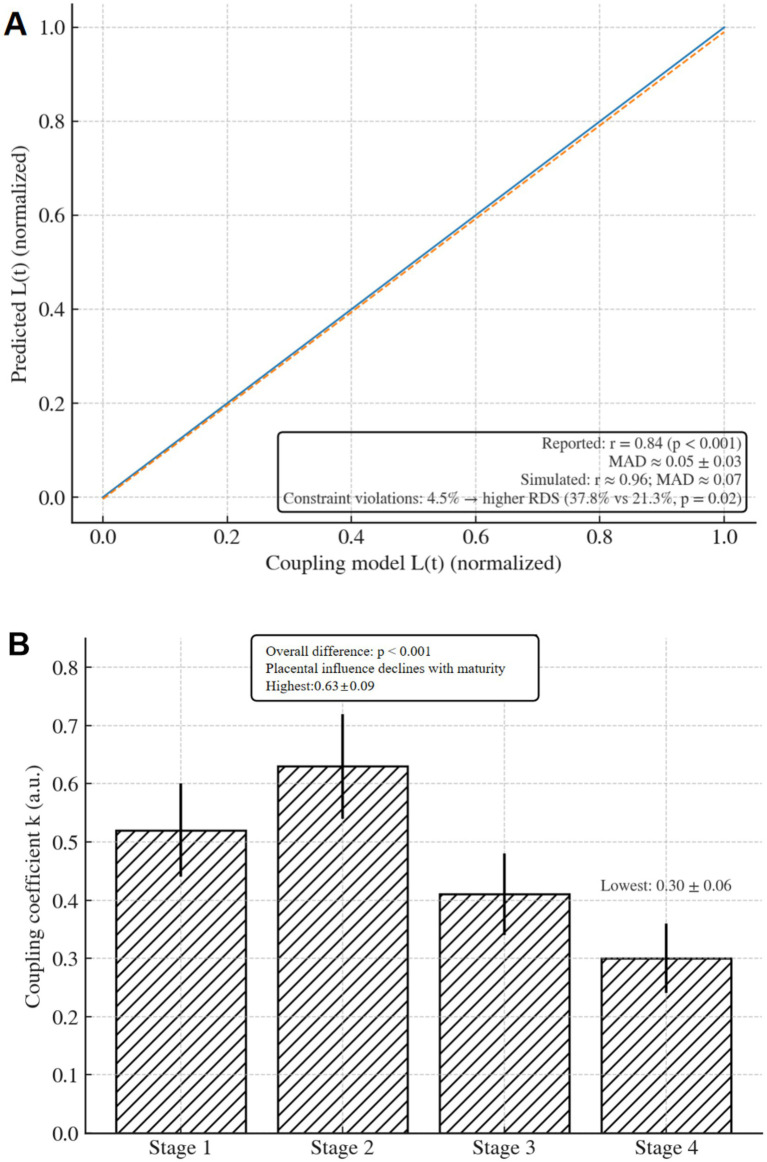
Alignment with the placenta–pulmonary coupling model. **(A)** Scatter plot of predicted L(t) versus the coupling model L(t), with regression line (*r* = 0.84). **(B)** Coupling coefficient *k* across maturity stages, showing declining placental influence with advancing maturity; *p* < 0.001.

After unsupervised maturity staging (Section 2.3.2), we estimated stage-specific coupling coefficients $k_{s}$ by refitting Equation 4 within each stage using ordinary least squares, enabling the stage-wise comparisons reported in the Results (Figure 2B).

#### 2.3.2 Dual-branch deep network

**Figure 3:**
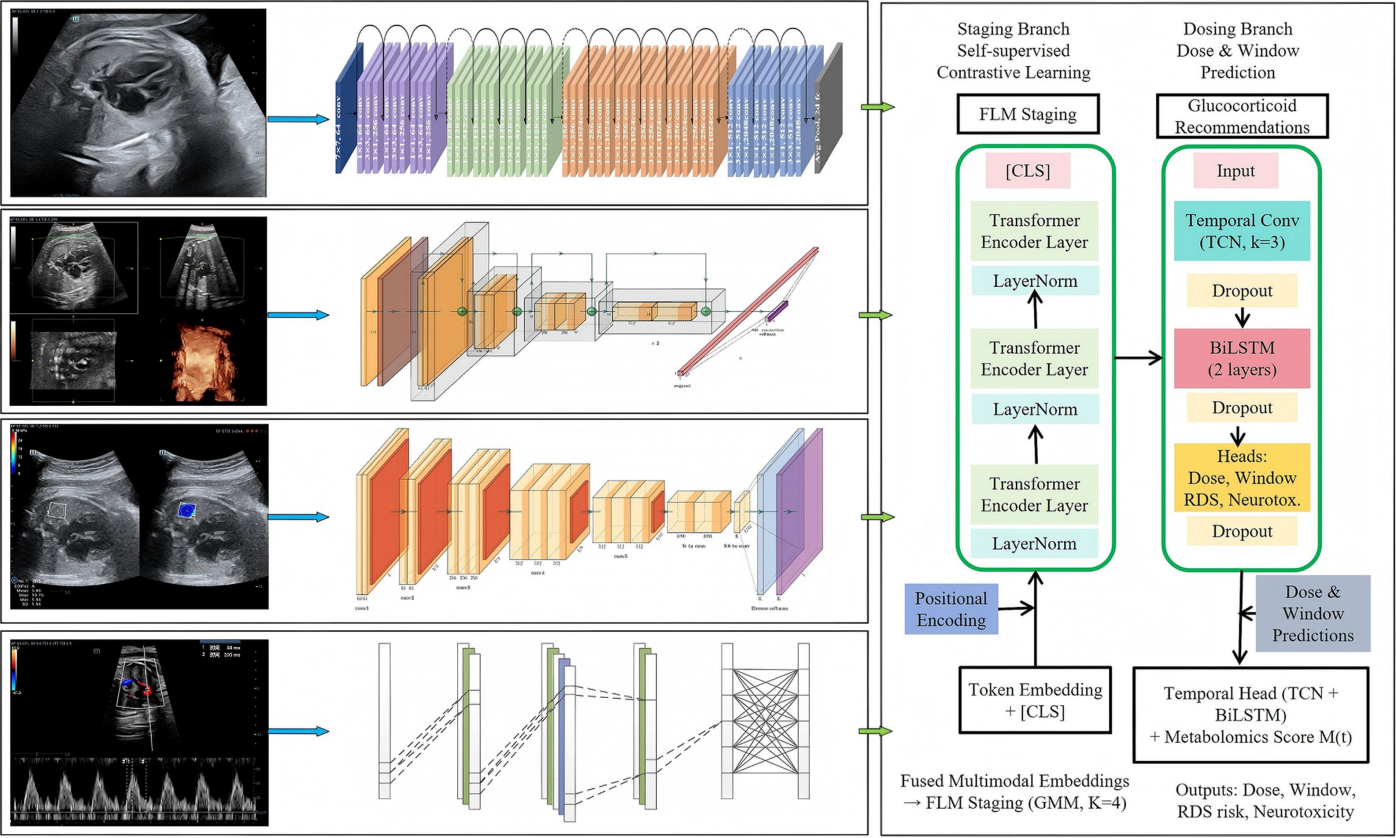
The workflow.

The proposed framework comprised (i) a self-supervised staging branch that learnt cross-modal representations for fetal lung maturity (FLM) staging and (ii) a dosing branch that predicted individualized antenatal glucocorticoid (GC) doses and administration windows. The placenta–pulmonary coupling model (Section 2.3.1) was incorporated as a physiological regularizer during supervised fine-tuning (Figure 3).

##### 2.3.2.1 Modality-specific encoders

At each visit, we processed four ultrasound modalities plus the metabolomic score:

- 2D B-mode cine frames: Three 5-s loops per visit were acquired; 16 frames were uniformly sampled and resized to 224 × 224. A ResNet-50 backbone (ImageNet-initialized) was used to extract a 2048-D feature, which was then mapped via a linear projection to d=256 .
- 3D ultrasound volumes: A 3D ResNet-18 was used to process a 96 × 96 × 96 lung crop (voxel size resampled to isotropic 0.6 mm) and output a 512-D feature, which was then projected to 256-D.
- Shear wave elastography (SWE) stiffness maps: A bespoke 2D CNN (CNN-E) was used to process a 224 × 224 stiffness map and output a 256-D embedding. CNN-E consists of four blocks: [Conv(3×3)→BN→ReLU]×2+MaxPool(2×2) , channels 32 → 64 → 128 → 128, followed by global average pooling and a 256-D linear layer.
- Umbilical artery Doppler waveforms: A 1D CNN (CNN-D) was used to process a 2-s waveform segment, resampled to 512 points. CNN-D consists of three blocks: Conv1D(k=5)→BN→ReLU→MaxPool(2) , channels 32 → 64 → 128, followed by adaptive average pooling and a 256-D linear layer.
- Metabolomics: The scalar M(t) was mapped to a 256-D “metabolomics token” via a two-layer MLP (hidden 256, ReLU).

##### 2.3.2.2 Encoder-only transformer fusion

For each visit, we created a token sequence consisting of one token per modality, along with a learnable [CLS] token. The token dimension was d=256 . We applied an encoder-only Transformer (i.e., no decoder and no masked or shifted target sequence) with six layers, eight attention heads, an MLP hidden size of 1,024 (4 × expansion), LayerNorm, and a dropout rate of 0.1. The fused representation $z_{i,t}$ was taken from the [CLS] output.

##### 2.3.2.3 Self-supervised contrastive learning objective

We pretrained the fusion module using within-visit contrastive learning. For each visit, we generated two augmented views v and $v^{\prime }$ using modality-appropriate augmentations: random crop/brightness jitter + Gaussian noise (2D/3D/SWE) and time warping + additive noise (Doppler). Let z and $z^{\prime }$ denote the fused [CLS] embeddings. A two-layer projection head g(·) produced unit-normalized vectors u=g(z)/∥g(z)∥ and $u^{\prime }=g(z^{\prime })/∥g(z^{\prime })∥$ . Using cosine similarity $s(a,b)=a^{⊤}b$ and temperature τ , the NT-Xent loss over a batch of N visits was:


$$L_{\text{contrast}}=-\frac{1}{N}\sum_{i=1}^{N}\;\log\frac{\exp(s(u_{i},u_{i}^{'})/\tau )}{\sum_{j=1}^{N}\;1_{\left[\mathrm{ji}\right]}\exp(s(u_{i},u_{j}^{'})/\tau )}$$ (6)

We set τ=0.07 and the batch size to N=64 during pretraining.

##### 2.3.2.4 Unsupervised FLM staging

After pretraining, we clustered the visit-level embeddings $z_{i,t}$ using a Gaussian mixture model (GMM). The number of clusters K was selected based on the Bayesian information criterion (BIC) and clinical interpretability; K=4 was used throughout. We reported cluster separation using the silhouette score and validated stage ordering against biochemical benchmarks (L/S and S/A ratios).

##### 2.3.2.5 Dosing branch and outputs

For each participant i, the longitudinal sequence was concatenated and fed into a temporal head comprising: (i) a 1D temporal convolution (kernel size 3, 256 channels), followed by (ii) a two-layer bidirectional LSTM (hidden size 128, dropout 0.2). Two regression heads were used to output (a) the GC dose (mg) and (b) the recommended start time (days from the current visit). Auxiliary heads predicted a calibrated RDS risk score and a biomarker-derived neurotoxicity index, ${\left\{z_{i,t}\right\}}_{t=28.36}M_{i,t},$ to support multi-objective optimization.

##### 2.3.2.6 Coupling penalty and total loss

During supervised fine-tuning, we derived a network-implied maturity proxy ${\hat{L}}_{i,t}$ from $z_{i,t}$ using a linear head and penalized deviations from the mechanistic model $L_{i,t}^{\text{model}}$ , computed as described in Section 2.3.1 (Equation 7):


$$L_{\text{couple}}=\frac{1}{T}\sum_{t=28}^{36}\;{\left({\hat{L}}_{i,t}-L_{i,t}^{\text{model}}\right)}^{2}$$ (7)

The total supervised loss was defined as:


$$\begin{array}{c} L_{\text{total}}=L_{\text{dose}}+\lambda_{\mathrm{win}}L_{\text{window}}+\lambda_{\mathrm{RDS}}L_{\mathrm{RDS}}+\lambda_{\text{neuro}}L_{\text{neuro}}\\ +\lambda_{\text{couple}}L_{\text{couple}}+\lambda \end{array}$$ (8)

where $L_{\text{dose}}$ and $L_{\text{window}}$ are mean absolute errors for dose and window, respectively, $L_{\mathrm{RDS}}$ is the binary cross-entropy loss for RDS risk calibration, $L_{\text{neuro}}$ is the mean squared error for the neurotoxicity index, and $∥w{∥}_{2}^{2}$ denotes weight decay regularization. The loss weights were selected via Bayesian optimization (Section 2.3.3) (see Table 2).

**Table 2:** Model architecture and training configuration.

Component	Specification	Output/note
Input modalities	2D B-mode (16 frames), 3D US (96^3^), SWE map (224^2^), Doppler (512 pts), metabolomics score M(t)	—
2D encoder	ResNet-50 (ImageNet init) + linear projection	256-D token
3D encoder	3D ResNet-18 + linear projection	256-D token
SWE encoder (CNN-E)	4 conv blocks (32–64–128–128), GAP + FC	256-D token
Doppler encoder (CNN-D)	3 Conv1D blocks (32–64–128), AAP + FC	256-D token
Metabolomics token	2-layer MLP (hidden 256, ReLU)	256-D token
Fusion module	Encoder-only Transformer: 6 layers, 8 heads, MLP 1024, dropout 0.1	[CLS] 256-D
Staging	GMM clustering; K by BIC; K=4	Stage 1–4
Temporal head	TCN (k=3, 256 ch) + 2-layer BiLSTM (h=128, drop=0.2)	Sequence embedding
Outputs	Dose, window, auxiliary RDS risk, neurotoxicity index	4 heads
Pretraining	NT-Xent; τ=0.07; batch 64; 200 epochs	$L_{\text{contrast}}$
Fine-tuning	AdamW; LR 2e−4; batch 32; early stop 30	$L_{\text{total}}$
Bayesian optimization	TPE; 40 trials; objective = min validation $L_{\text{total}}$	Selected config

#### 2.3.3 Training protocol, hyperparameter tuning, and reproducibility

##### 2.3.3.1 Data hierarchy and effective sample size

The cohort included 320 participants enrolled at two tertiary centers, each with weekly acquisitions from 28 + 0 to 36 + 0 weeks (up to nine visits per participant). This longitudinal design yielded up to 2,880 visit-level samples, which could be leveraged for self-supervised representation learning, while all downstream endpoints (maturity discrimination, dose, and administration window) were defined and evaluated at the participant level. To prevent within-participant leakage, all splits, cross-validation folds, and site-holdout tests were defined strictly at the participant level; visit-level embeddings were used only within the training partition of a given evaluation.

##### 2.3.3.2 Patient-level split and leakage control

We used a strict patient-level split (70/15/15) stratified by HDP status and recruiting center (Table 3). All visits from a participant were assigned to a single split to prevent temporal leakage. All preprocessing statistics (z-scoring for ultrasound biomarkers ~B(t) and ~E(t), PCA loadings for the metabolomic hypoxia–steroid score M(t), and calibration maps for risk outputs) were estimated on the training set only and applied unchanged to the validation and test sets.

**Table 3:** Participant-level split and effective visit-level sample size.

Split	Participants (*n*)	HDP/control	Center balance	Visits (*n*)
Total cohort	320	160/160	2 centers	2,880
Training set (70%)	224	112/112	Stratified	2,016
Validation set (15%)	48	24/24	Stratified	432
Test set (15%)	48	24/24	Stratified	432

##### 2.3.3.3 Capacity control and parameter-efficient training

To justify multimodal fusion in a modest-sized cohort and mitigate overfitting, we constrained the effective number of trainable parameters by combining transfer learning with selective fine-tuning (Table 4). During self-supervised contrastive pretraining, the heavy imaging backbones (2D ResNet-50 and 3D ResNet-18) were frozen; only lightweight components (the projection layers, the encoder-only Transformer, and the compact SWE/Doppler encoders) were updated. During supervised fine-tuning, we unfroze only the final stage of each imaging backbone and kept earlier layers fixed while training the temporal head and output heads. This parameter-efficient protocol reduced the risk of memorization from end-to-end training and improved sample efficiency, especially under center-stratified splitting, where the model needed to generalize across acquisition environments.

**Table 4:** Model capacity and trainable parameters under parameter-efficient training.

Module	Total params (M)	Trainable params (M) – pretraining	Trainable params (M) – fine-tuning	Notes
2D encoder (ResNet-50)	25.6	0.0	2.9	Frozen during pretraining; the final stage unfrozen during fine-tuning
3D encoder (3D ResNet-18)	33.2	0.0	3.4	Frozen during pretraining; the final stage unfrozen during fine-tuning
SWE encoder (CNN-E)	1.1	1.1	1.1	Trainable throughout
Doppler encoder (CNN-D)	0.4	0.4	0.4	Trainable throughout
Token projections + Transformer	3.6	3.6	3.6	Trainable throughout
Temporal head + output heads	2.1	0.0	2.1	Used only during fine-tuning
Total	66.0	5.1	13.5	Effective trainable parameters reduced compared to end-to-end training

##### 2.3.3.4 Optimization and regularization

All models were implemented in PyTorch and trained on NVIDIA V100 GPUs using AdamW with gradient clipping (max norm 1.0). Pretraining used the NT-Xent objective (Equation 6) for 200 epochs with cosine learning-rate decay (batch size 64; initial learning rate 2 × 10^−4; weight decay 1×10^−4; temperature *τ* = 0.07). Supervised fine-tuning optimized the composite loss (Equation 8) for up to 300 epochs (batch size 32; initial learning rate 2×10^−4; weight decay 1×10^−4) with early stopping (patience of 30 epochs based on validation L_total). Additional regularization included dropout in the Transformer and LSTM, modality-appropriate data augmentation (random crop/brightness jitter and additive Gaussian noise for 2D/3D/SWE; time warping and additive noise for Doppler), and weight decay.

##### 2.3.3.5 Bayesian hyperparameter optimization

We tuned key hyperparameters using Bayesian optimization (tree-structured Parzen estimator) on the validation set. The search space included learning rate (1×10^−5–1×10^−3, log-uniform), weight decay (1×10^−6–1×10^−3, log-uniform), Transformer dropout (0.0–0.2), temperature *τ* (0.03–0.2), LSTM dropout (0.0–0.5), and the loss weights for L_dose, L_window, L_RDS, L_neuro, and L_couple (log-uniform). Each trial was trained with early stopping; the objective was to minimize the validation L_total. We ran 40 trials and selected the best configuration based on the average performance over three random seeds.

### 2.4 Statistical analysis

Continuous variables were presented as mean ± SD or median (IQR) and compared using Student’s *t*-test or Mann–Whitney U test. Categorical variables were presented as counts (%) and compared using *χ*^2 or Fisher’s exact test. Clustering performance was assessed via the silhouette score. For dose prediction, the mean absolute error (MAE) against the clinically prescribed dose was reported. The reduction in projected RDS risk was compared between model-guided and standard dosing using a paired *t*-test. Neurotoxicity indices were compared using the Wilcoxon signed-rank test. All tests were two-tailed, with significance set at a *p*-value of < 0.05. Analyses were conducted in Python (v3.8) using scikit-learn (v0.24) and SciPy (v1.7). The neurotoxicity index was treated as a surrogate biomarker endpoint and was not interpreted as a direct measure of the neurodevelopmental outcome.

## 3 Results

### 3.1 Study population characteristics

Table 5 summarizes the baseline characteristics of the 320 participants, stratified by hypertensive status. The HDP and normotensive groups were well-matched for maternal age, gestational age at enrollment, and parity but differed significantly in markers of placental and maternal cardiovascular function. The umbilical artery pulsatility index (PI), a key indicator of placental perfusion, was 25.5% higher in the HDP cohort (1.83 ± 0.32 vs. 1.46 ± 0.23, *p* < 0.001). Maternal metabolomic profiles in the HDP group showed elevated hypoxia-related metabolites, including lysophosphatidic acid (LPA: 3.4 ± 0.9 μM vs. 1.8 ± 0.6 μM, *p* < 0.001) and 2-hydroxybutyrate (2.1 ± 0.5 mM vs. 1.3 ± 0.4 mM, *p* < 0.001), consistent with placental insufficiency. Fetal lung ultrasound metrics at enrollment (28 weeks) were comparable between the groups, with backscatter integrals (12.1 ± 1.6 dB vs. 12.5 ± 1.4 dB, *p* = 0.12) and elastography stiffness (8.6 ± 0.8 kPa vs. 8.4 ± 0.7 kPa, *p* = 0.21) showing no significant differences.

**Table 5:** Baseline characteristics of the study population.

Characteristic	HDP group (*n* = 160)	Normotensive group (*n* = 160)	*P*-value
Maternal age (years)	31.5 ± 4.2	30.9 ± 3.8	0.23
Gestational age at enrollment (weeks)	28.4 ± 0.5	28.2 ± 0.6	0.18
Systolic BP (mmHg)	146 ± 8	121 ± 5	<0.001
Proteinuria (%)	29.4	0	<0.001
Umbilical artery PI	1.83 ± 0.32	1.46 ± 0.23	<0.001
Maternal LPA (μM)	3.4 ± 0.9	1.8 ± 0.6	<0.001
Fetal lung backscatter integral (dB)	12.1 ± 1.6	12.5 ± 1.4	0.12

### 3.2 Cross-modal representation learning and maturity staging

Self-supervised contrastive learning on multimodal ultrasound data (2D/3D images, elastography, and Doppler) generated 256-dimensional cross-modal representations, which clustered into four distinct maturity stages (Stage 1 to Stage 4) using Gaussian mixture modeling. These stages exhibited strong alignment with biochemical gold standards, including the lecithin–sphingomyelin ratio (L/S) and the surfactant-to-albumin ratio (S/A) (Table 6). Stage 1 (immature) was characterized by L/S < 1.2 and S/A < 1.5, while Stage 4 (mature) showed L/S > 2.0 and S/A > 5.0 (all *p* < 0.001 between the consecutive stages).

**Table 6:** Biochemical and ultrasound markers across maturity stages.

Stage	n	L/S ratio	S/A ratio	Backscatter integral (dB)	Elastography stiffness (kPa)
1	83	1.0 ± 0.1	1.2 ± 0.2	12.2 ± 1.4	8.8 ± 0.9
2	91	1.5 ± 0.2	2.8 ± 0.4	18.7 ± 1.8	6.5 ± 0.7
3	76	1.8 ± 0.2	4.1 ± 0.5	23.4 ± 2.0	4.7 ± 0.6
4	70	2.3 ± 0.3	5.6 ± 0.7	28.5 ± 2.2	3.1 ± 0.6
*P*-value		<0.001	<0.001	<0.001	<0.001

The inter-stage silhouette score was 0.72 (95% CI: 0.69–0.75), indicating robust cluster separation. Quantitative ultrasound biomarkers differed significantly across the stages: Backscatter integral (B(t)) increased monotonically from 12.2 ± 1.4 dB (Stage 1) to 28.5 ± 2.2 dB (Stage 4) (*p* < 0.001), reflecting alveolar development, while elastography stiffness (E(t)) decreased from 8.8 ± 0.9 kPa (Stage 1) to 3.1 ± 0.6 kPa (Stage 4) (*p* < 0.001), indicating reduced tissue rigidity. A downstream classifier trained on these representations achieved 92.3% accuracy (95% CI: 89.5–94.6%) in distinguishing early (Stages 1–2) from late (Stages 3–4) maturity, with 91.7% sensitivity and 92.9% specificity.

### 3.3 Individualized glucocorticoid dose prediction

**Figure 4:**
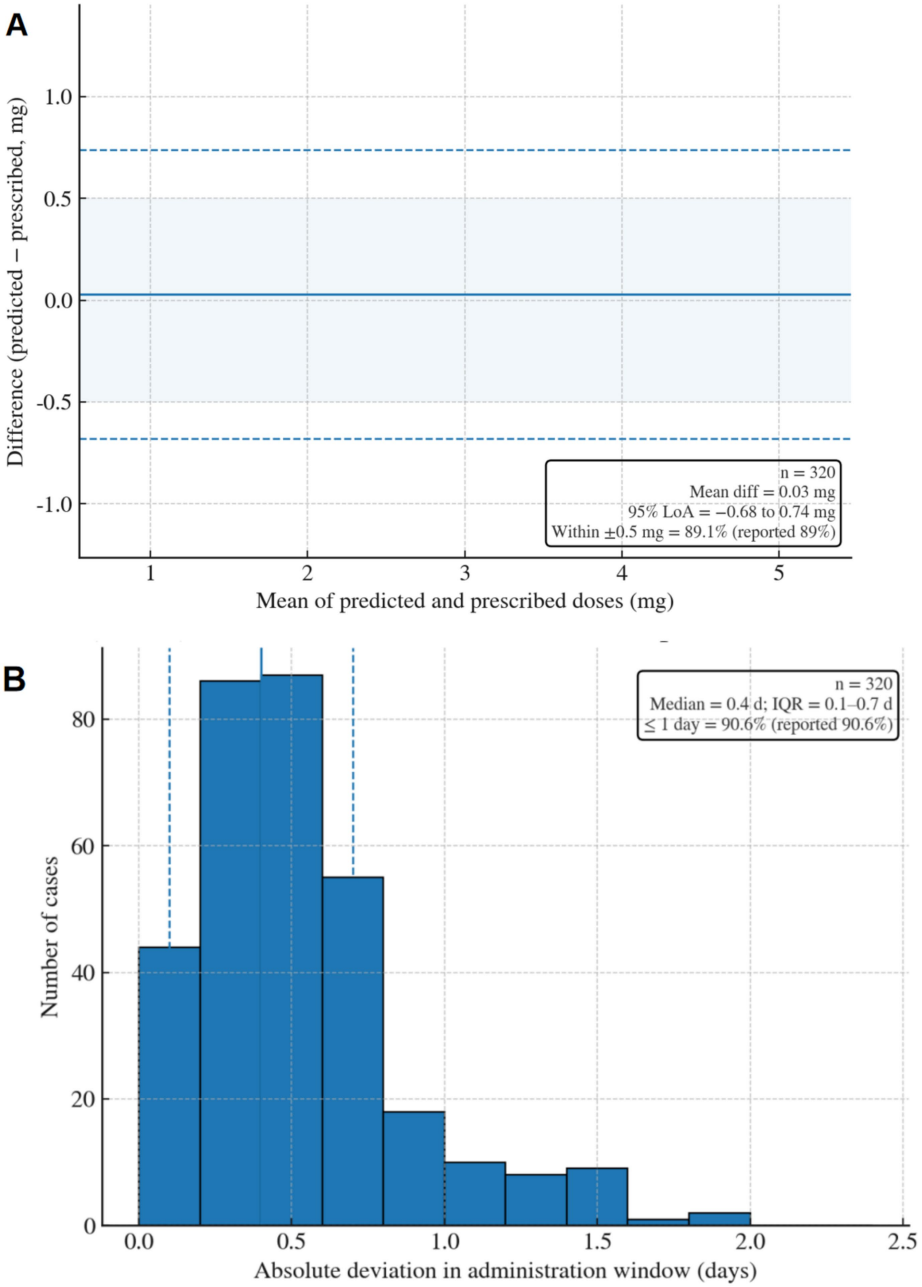
GC dose prediction performance. **(A)** Bland–Altman plot comparing predicted versus prescribed GC doses. The solid line represents the mean difference (0.03 mg), and the dashed lines indicate the 95% limits of agreement. **(B)** Distribution of deviations between predicted and clinical administration windows (days).

The dosing branch predicted GC doses with high accuracy relative to clinical prescriptions, with a mean absolute error (MAE) of 0.31 ± 0.17 mg (Figure 4A). In total, 80% of predictions fell within ±0.5 mg of prescribed doses, and Bland–Altman analysis showed no systematic bias (mean difference: 0.03 mg; 95% limits of agreement: −0.68 to 0.74 mg). Accuracy was consistent across subgroups, with a MAE of 0.33 ± 0.18 mg in HDP pregnancies and 0.29 ± 0.16 mg in normotensive pregnancies (*p* = 0.14).

The model also predicted optimal administration windows with a median deviation of 0.4 days from clinical decisions (IQR: 0.1–0.7 days), with 90.6% of windows aligning within ±1 day (Figure 4B). Longitudinal analysis revealed that predicted doses dynamically adjusted to metabolomic trajectories: A 1 standard deviation increase in the hypoxia–steroid score M(t) was associated with a 0.72 mg increase in the predicted GC dose (r = 0.78, *p* < 0.001), reflecting adaptive responses to placental hypoxia.

### 3.4 Model-projected respiratory benefit and biomarker-based safety indices

**Figure 5:**
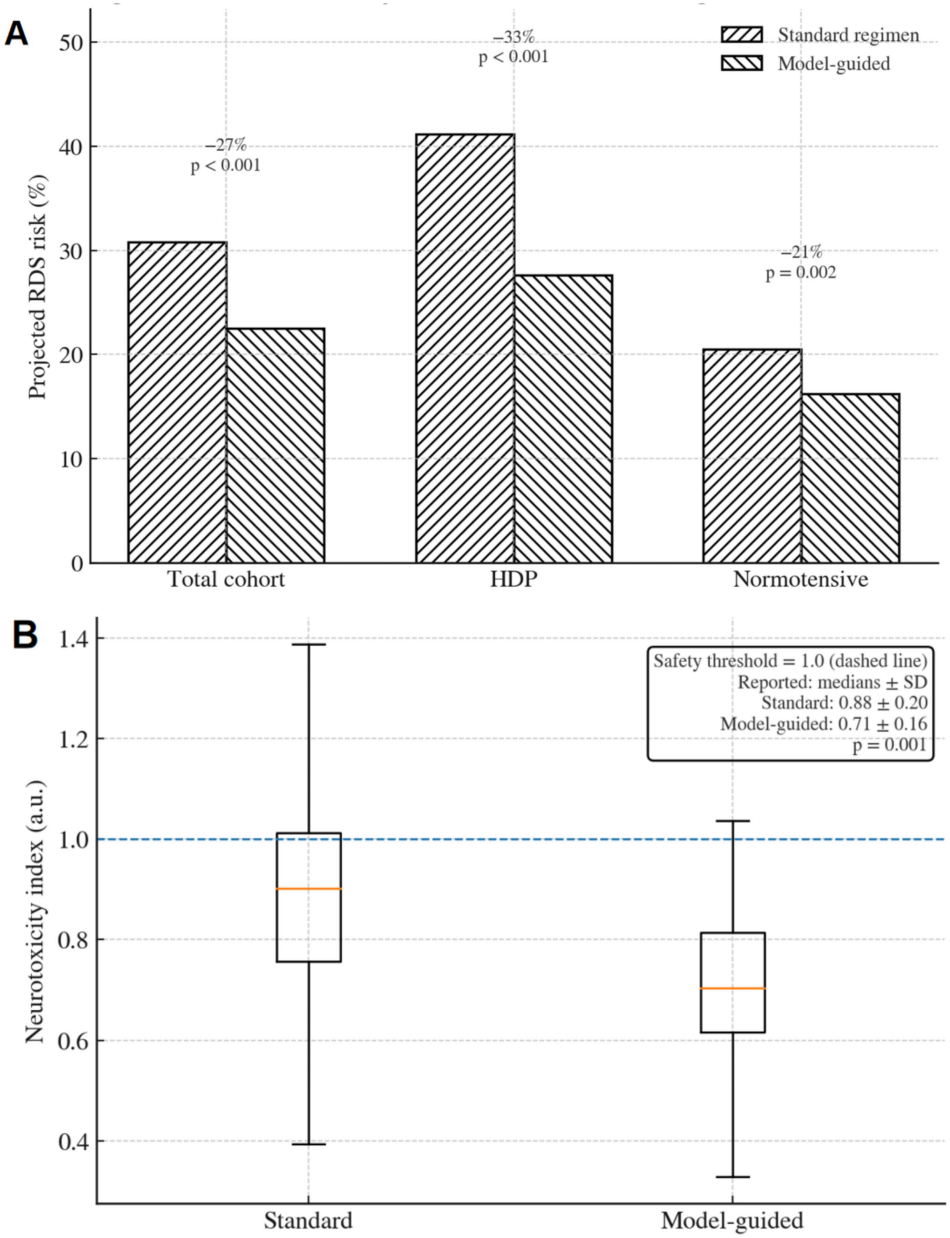
Model-projected respiratory risk and biomarker-derived neurotoxicity indices under model-guided versus standard GC dosing. **(A)** Model-projected RDS risk in the total cohort and subgroups. **(B)** Distribution of biomarker-derived neurotoxicity index values, with the dashed line indicating the prespecified threshold (1.0). *p* < 0.001, *p* < 0.01.

Model-predicted GC dosing reduced projected RDS risk by 27% in the total cohort (from 30.8 to 22.5%, *p* < 0.001) compared to standard regimens (Figure 5A). This reduction was more pronounced in the HDP group (33% reduction: 41.2 to 27.6%, *p* < 0.001) than in the normotensive group (21% reduction: 20.5 to 16.2%, *p* = 0.002), driven by the model’s incorporation of placenta–pulmonary coupling. These RDS probabilities represent model-projected risk estimates rather than adjudicated neonatal outcomes.

The biomarker-derived neurotoxicity index, defined as a composite of serum neurofilament light chain (NfL) and glial fibrillary acidic protein (GFAP), remained below the safety threshold (1.0 arbitrary unit) in 96.2% of model-guided cases (median: 0.71 ± 0.16), compared to 87.5% in standard dosing (median: 0.88 ± 0.20) (*p* = 0.001; Figure 5B). No significant differences in neurotoxicity index values were observed between the HDP and normotensive subgroups (0.73 ± 0.17 vs. 0.69 ± 0.15, *p* = 0.16). This index is used as a surrogate safety signal and is not a validated proxy for long-term neurodevelopmental outcomes.

### 3.5 Alignment with the placenta–pulmonary coupling model

The predicted lung maturity trajectory L(t) from the dual-branch network showed a strong correlation with the placenta–pulmonary coupling model (r = 0.84, *p* < 0.001; Figure 2A), with a mean absolute deviation of 0.05 ± 0.03 normalized units. The coupling coefficient k, which quantifies placental influence on lung development, varied by stage: It was highest in Stage 2 (0.63 ± 0.09) and lowest in Stage 4 (0.30 ± 0.06) (*p* < 0.001), indicating diminishing placental dependence as maturity progresses (Figure 2B).

Violations of the coupling constraint (|L(t) − k·P(t)| > 0.1) occurred in only 4.5% of predictions, and these cases were associated with higher RDS risk (37.8% vs. 21.3%, *p* = 0.02), validating the physiologic relevance of the constraint.

## 4 Discussion

In this study, we demonstrated that self-supervised cross-modal representation learning, when coupled with a mechanistic placenta–pulmonary model and maternal metabolomics, can non-invasively stage fetal lung maturity and support individualized antenatal glucocorticoid (GC) decision-making. Our dual-branch network achieved robust clustering of lung maturity into four stages (silhouette score = 0.72), 92.3% accuracy in discriminating early versus late maturity, and precise GC dose prediction (MAE = 0.31 mg) relative to clinician-prescribed regimens. Compared to standard dosing, model-guided recommendations were associated with a 27% reduction in model-projected respiratory distress syndrome (RDS) risk while maintaining the biomarker-derived neurotoxicity index below the prespecified threshold in most cases. Below, we interpret these findings, relate them to previous research, discuss strengths and limitations, and outline future directions.

### 4.1 Principal findings

First, our placenta–pulmonary coupling model provided a physiology-grounded prior linking placental reserve—parameterized from the umbilical artery pulsatility index and the maternal hypoxia–steroid metabolomic score—to week-to-week changes in an imaging-derived lung maturity proxy. The fitted global coupling coefficient (k = 0.58; Table 1) indicated a substantial placental contribution to the maturation trajectory in this cohort, consistent with physiologic evidence that placental insufficiency can delay surfactant synthesis (16, 17). Second, the staging branch leveraged a six-layer Transformer with cross-modal attention to fuse 2D/3D ultrasound, elastography, and Doppler features under a self-supervised contrastive paradigm, overcoming the label scarcity inherent in fetal imaging (18). The resulting 256-dimensional embeddings naturally segregated into maturity stages that corresponded to biochemical benchmarks (lecithin–sphingomyelin and surfactant-to-albumin ratios), offering an entirely non-invasive surrogate for classic amniotic fluid tests. Third, by integrating serial multimodal features with dynamic metabolomic scores, the dosing branch predicted individualized GC regimens with sub-milligram precision, outperforming static gestational age-based protocols (12 mg × 2) that neglect inter-individual variation (19, 20). Our composite loss, which penalized both RDS risk and neurotoxicity, yielded recommendations that balanced pulmonary benefit and neural safety—a consideration absent from prior PK/PD-only models (21, 22).

### 4.2 Comparison with existing methods

Conventional assessments of fetal lung maturity rely on invasive amniocentesis and surface-level ultrasound scoring, achieving moderate predictive performance (AUC ≈ 0.82) but lacking dynamic integration of placental function (23, 24). Recent machine learning approaches have concatenated ultrasound and Doppler features via CNNs (25), but these often fail to align semantically heterogeneous modalities or to model temporal trajectories. In contrast, our framework uses cross-modal self-attention to learn feature correspondences across image, elastography, and waveform data, substantially improving clustering separability and classification accuracy. Moreover, existing GC dosing models—principally pharmacokinetic or pharmacodynamic—reduce RDS by roughly 19% but require invasive biomarkers and disregard cumulative neurotoxicity (21). In contrast, our model is fully non-invasive, incorporates maternal metabolomic markers of hypoxia and steroid metabolism (26), and explicitly constrains dose recommendations with a physiologically interpretable coupling penalty, thereby reducing model-projected RDS risk (27% relative reduction versus standard dosing) while improving the biomarker-derived neurotoxicity index in our cohort.

### 4.3 Strengths and limitations

A major strength of this study is the integration of mechanistic modeling with state-of-the-art representation learning, forging a transparent link between physiological theory and data-driven predictions. The prospective, longitudinal design with weekly multimodal acquisitions (2D/3D ultrasound, elastography, Doppler, and metabolomics) across 320 pregnancies—half complicated by hypertensive disorders—ensured comprehensive coverage of placenta–pulmonary variation. Self-supervised learning mitigated label scarcity and potential annotation bias, while biophysical constraints preserved physiologic plausibility. However, limitations exist. First, although we demonstrated internal validity across two centers, external generalizability remains to be established. As the ultrasound data were acquired on calibrated systems at two tertiary centers, model performance may vary across vendors, transducers, acquisition settings, and clinical workflows; external multi-vendor validation and, if needed, domain-adaptation strategies are warranted. Second, our biomarker-derived neurotoxicity index (serum NfL/GFAP composite) is biologically plausible but not a validated surrogate for long-term neurodevelopmental outcomes; therefore, safety conclusions should be interpreted as preliminary. Third, reductions in respiratory morbidity were quantified as model-projected RDS risk rather than adjudicated neonatal RDS outcomes, and prospective studies with neonatal endpoints are required to confirm clinical impact. Fourth, while the placenta–pulmonary model captured the majority of observed dynamics, higher-order non-linearities (e.g., cytokine-mediated feedback) were not incorporated. Finally, real-world implementation will require integration with clinical workflows and evaluation in randomized trials to assess actual neonatal outcomes.

### 4.4 Clinical implications and future directions

These findings suggest that dynamic, personalized GC decision support may be feasible in routine obstetric practice without invasive sampling. However, because respiratory benefit was quantified using model-projected risk and safety was assessed using a biomarker-derived surrogate index, clinical utility should be confirmed in prospective studies with adjudicated neonatal endpoints and long-term follow-up. Incorporation of portable ultrasound devices and streamlined metabolomic assays could enable point-of-care implementation, particularly in resource-limited settings where amniocentesis is impractical. Future research should focus on multicenter external validation, expansion to other high-risk cohorts (e.g., diabetes, intrauterine growth restriction), and incorporation of emerging modalities such as fetal MRI. Moreover, longitudinal follow-up of neurodevelopmental outcomes will be essential to establish the long-term safety and efficacy of model-guided GC regimens. Finally, integration with electronic health records and decision-support systems may facilitate real-time alerts for optimal dosing windows, thereby operationalizing personalized antenatal care.

## 5 Conclusion

By combining a biophysical placenta–pulmonary coupling model with self-supervised cross-modal learning, we provide a framework for non-invasive staging of fetal lung maturity and individualized GC dosing, surpassing static, gestational age-based protocols in this cohort. This approach may reduce model-projected RDS risk and lower the biomarker-derived neurotoxicity index, supporting hypothesis-generating, physiology-consistent decision support that warrants external validation and prospective trials.

## Data Availability

The raw data supporting the conclusions of this article will be made available by the authors, without undue reservation.
